# The Influence of Tobacco Smoking Intensity on Hemodynamic Parameters: A Functional Transcranial Doppler Study on Vascular Reserve in Chronic Smokers

**DOI:** 10.7759/cureus.72956

**Published:** 2024-11-04

**Authors:** Sandra Lakicevic, Marija Bender, Goran Lakicevic, Renata Jurina, Nina Mihic, Branko Malojcic

**Affiliations:** 1 Clinic of Neurology, University Clinical Hospital Mostar, Mostar, BIH; 2 Clinic of Neurosurgery, University Clinical Hospital Mostar, Mostar, BIH; 3 Faculty of Health Studies, Institute of Health Insurance of Herzegovina-Neretva County, Mostar, BIH; 4 Clinic of Neurology, University Hospital Center Zagreb, Zagreb, HRV

**Keywords:** chronic smokers, functional transcranial doppler, neurovascular response, tobacco smoking, vascular reserve

## Abstract

Objective: Tobacco smoking is an independent risk factor for stroke. In acute and chronic settings, it affects cerebral blood flow, mean systolic velocities, changes of velocities in response to metabolic challenges, pulsatility, and resistance indices in otherwise healthy smokers. The objective of the study was to determine the influence of smoking intensity on hemodynamic parameters in nonsmokers and smokers.

Methods: This prospective study enrolled 34 healthy volunteers, 19 smokers and 15 nonsmokers. Epidemiological data were taken from all patients, and exhaled carbon monoxide concentration was measured in smokers. To obtain hemodynamic parameters, we performed functional transcranial Doppler (fTCD) recordings on the posterior cerebral artery for six minutes, consisting of three cycles with closed and opened eyes. We investigated mean systolic velocities, neurovascular responses, and pulsatility indices.

Results: Smokers had significantly lower blood flow velocities than nonsmokers (28.36±5.87 and 30.19±6.41, respectively). Neurovascular response as a marker of vasodilatory potential was significantly lower in smokers (14.27%±0.08%) than in the nonsmoker group (17.33%±0.06%). Smokers with higher exhaled carbon monoxide concentrations had lower blood flow velocities than those with lower CO concentrations. Smokers had higher levels of pulsatility indices compared to nonsmokers.

Conclusion: The vasodilatory mechanism of cerebral blood vessels is impaired in chronic, otherwise healthy smokers.

## Introduction

Tobacco smoking is an independent risk factor for different types of stroke. The immediate effect of one smoked cigarette leads to an increase in heart rate and mean arterial pressure and a decrease in pulsatility index (PI), while long-term effects have an impact on atherosclerosis acceleration [1]. The risk increases proportionally to the number of cigarettes smoked and the duration of smoking [2,3].

The neurovascular response represents vasodilatation of brain resistance vessels that serves as a protective mechanism for stable and adequate perfusion for metabolic activities. Functional transcranial Doppler offers the possibility of evaluating rapid changes in hemodynamic parameters with excellent temporal resolution. Because the visual cortex is almost exclusively supplied with blood from the posterior cerebral artery, visual stimulation is reliable for assessing the neurovascular response.

Pathological changes in the neurovascular response seen after ischemic stroke, migraine, amyloid angiopathy, and dementia are underinvestigated in chronic smokers [4-7]. There is especially no data on the influence of intensity of smoking on hemodynamic parameters. Henceforth, here we present a prospective study aimed at investigating the same parameters in chronic smokers and their possible change in relation to the intensity of smoking.

## Materials and methods

The research was conducted prospectively over a three-month period at the Neurology Outpatient Clinic of University Clinical Hospital Mostar in Mostar, Bosnia and Herzegovina. We enrolled 34 healthy volunteers, 19 smokers and 15 nonsmokers. The participants were examined for complaints that did not affect the results of this research, such as low back pain, neck pain, peripheral vertigo, headaches, and epilepsy outside of attacks. Patients consuming electronic cigarettes were excluded from this study.

All participants were included on a voluntary basis and signed an informed consent form. Approval from the Bioethical Commission of the University Clinical Hospital Mostar has been obtained under the number 2947/15. 

All subjects were subjected to measurement of exhaled carbon monoxide (CO) concentration with a piCO simple Smokerlyzer device (Bedfont Scientific Ltd, Harrietsham, England). The reading was expressed in ppm (number of CO molecules in a million air particles). Depending on the concentration of CO [8], the subjects were classified into the group of nonsmokers (0-6 ppm) or smokers, who were stratified into moderate smokers (11-15 ppm), frequent smokers (16-25 ppm), regular smokers (25-35 ppm), heavy smokers (36-50 ppm), and very heavy smokers (51+ ppm). 

After classifying the patients, they were asked to complete a questionnaire with general epidemiological data. They were advised to refrain from smoking for at least 3-4 hours to avoid the acute effect of smoking on the measured parameters. Patients consuming electronic cigarettes were excluded from this study.

Ultrasound imaging protocol

**Figure 1:**
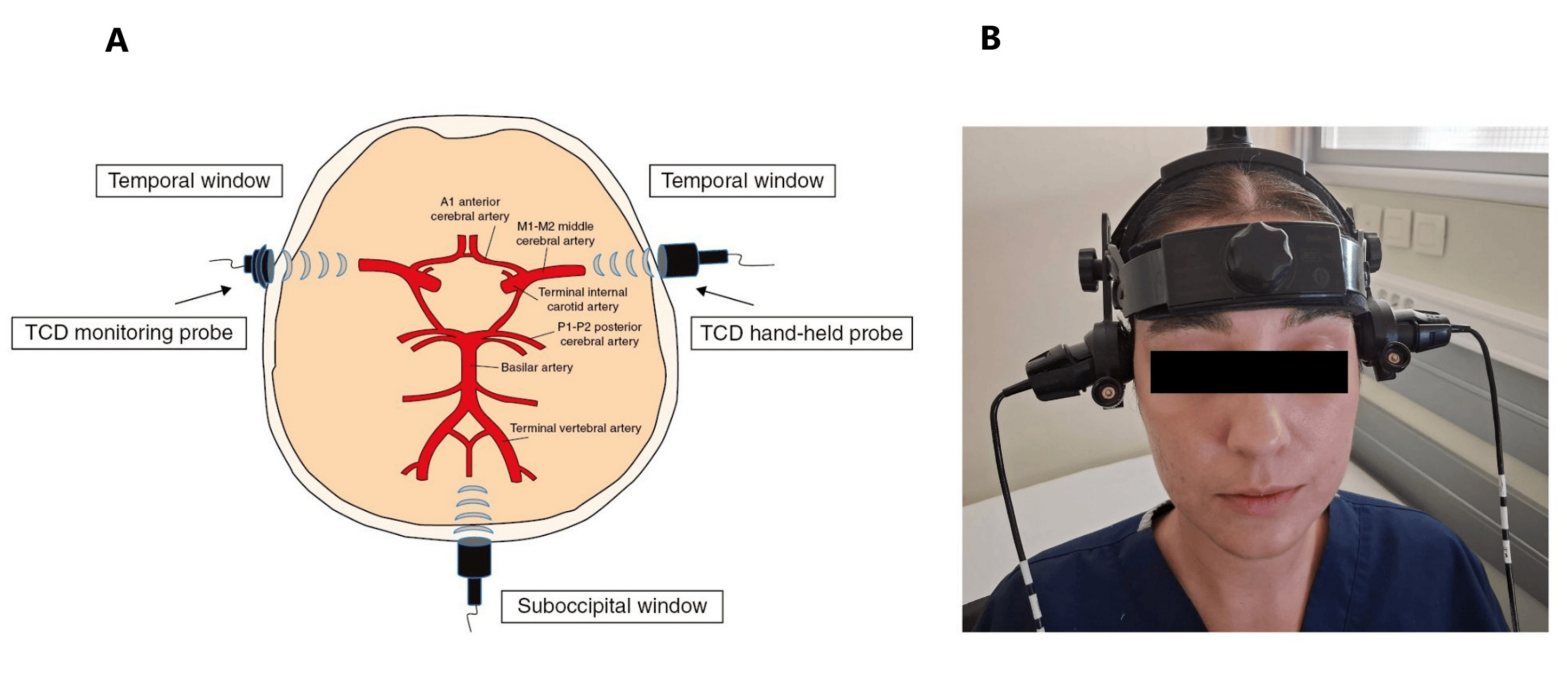
(A) Ultrasound bone window approaches to the circle of Willis with routine scanning or monitoring probes. (B) TCD monitoring probes being held in place with a headband TCD: transcranial Doppler

The hemodynamic parameters were measured in the evening using a transcranial Doppler BoxX device (DWL, Singen, Germany) (Figure 1).

**Figure 2:**
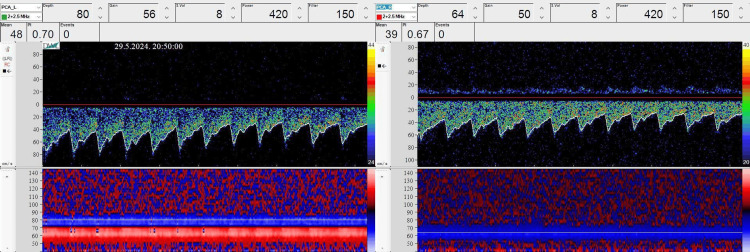
View of the hemodynamic spectrum of the left and right posterior cerebral arteries

Both posterior cerebral arteries were insonated through the transtemporal bone windows using 2 MHz probes attached by a special frame in the position of the best signal. We insonated the P2 segments of posterior cerebral arteries according to standardized protocols [9] (Figure 2).

The entire measurement was recorded using a continuous flow monitoring application (two-channel bilateral) that automatically registers changes in mean blood flow velocities (MFVs) and PI and shows their trends in time.

**Figure 3:**
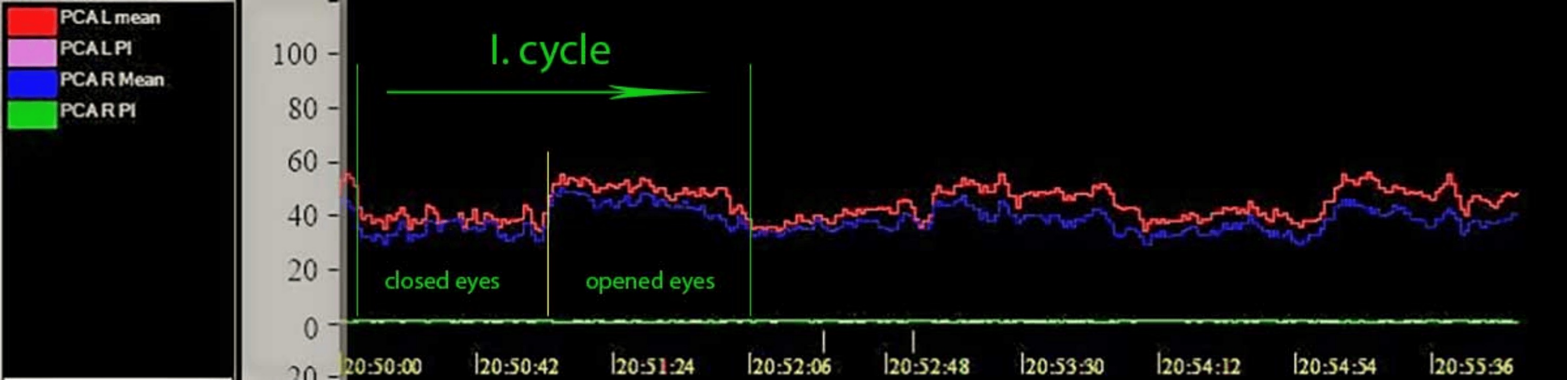
Trends in mean blood flow velocities across three cycles (eyes closed/opened) in the left and right posterior cerebral arteries

Patients were insonated in a comfortable, semi-sitting position in a dark and quiet room. After the adjustment phase, a six-minute recording consisting of three cycles was made. Each cycle consisted of an eyes-closed phase (rest) lasting one minute and an eyes-opened phase (stimulation) lasting one minute (Figure 3).

In the stimulation phase, the patients read an emotionally neutral text on the computer screen, which represented a visual paradigm. A sound signal indicated the transition from one cycle phase to another. The neurovascular response represented the percentage change in velocities during the eyes-opened phase (stimulation) compared to the last 10 seconds of the eyes-closed phase (rest) and was calculated using the following formula: $dV_{Left_/ Right} \text{(t)(\%)}=100 \ast \frac{V_{Left_/ Right} \text{(t)}-V_{pre.mean,Left_/ Right}}{V_{pre.mean,Left_/ Right}}$. Here, $V_{Left_/ Right} \text{(t)}$ represented the average blood flow velocity for the left or right side, calculated from all three stimulations, $V_{pre.mean,Left_/ Right}$ represented the average blood flow velocity for the last 10 seconds of the eyes-closed period, and $(t)$ is the duration of the visual paradigm in seconds.

The sampling time of MFV and PI during recording was set to one second. A total of 48,960 samples were exported to an Excel program and analyzed offline. The software system R Core Team (2023) R: A Language and Environment for Statistical Computing (R Foundation for Statistical Computing, Vienna, Austria (https://www.R-project.org/)) was used for the statistical analysis and graphical display of data. Microsoft Excel (Version 16.89.1, Microsoft Corporation, Redmond, Washington, United States) was also used for data wrangling. The tests used were the one-tailed t-test and the analysis of variance (ANOVA) test. A p-value of <0.05 is considered statistically significant, but p-values less than 0.10 were also commented in the article.

## Results

After analyzing the frequency distribution among the groups, we noted a higher proportion of male participants in the smoker group (p=0.04). No statistically significant differences were found in other variables such as age, marital status, education, place of residence, social status, or household smoking. The majority of smokers were classified as moderate and frequent smokers, with an average age of 35±2 years.

Influence of smoking on hemodynamic parameters

**Figure 4:**
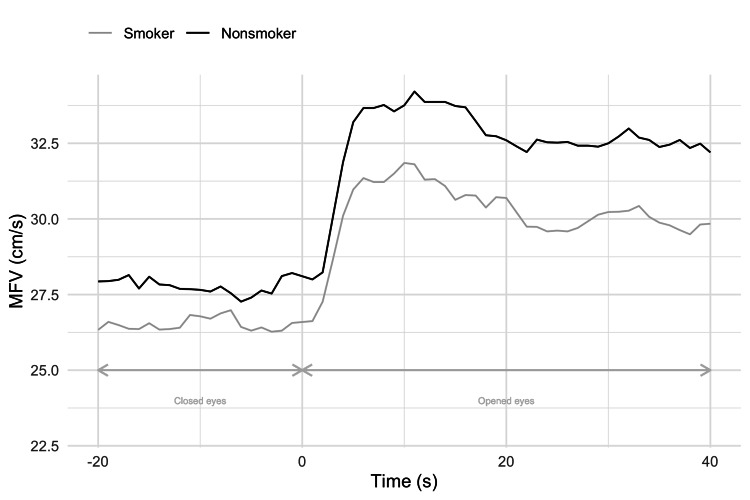
Total MFV according to phases in nonsmokers and smokers MFV: mean blood flow velocity

MFVs were measured in the last 20 seconds of the eyes-closed phase and the first 40 seconds of the eyes-opened phase in all three cycles bilaterally. The total result was formed as the average value of MFV in all three cycles on both sides. A parametric one-tailed t-test used to examine differences showed that smokers had significantly lower cerebral blood flow (CBF) compared to nonsmokers, at a significance level of 15% (Figure 4).

MFVs in smokers and nonsmokers were analyzed independently of phases. The results, presented as the average MFV value over the entire measurement, showed a significant difference using a t-test. Higher MFVs were demonstrated in nonsmokers (30.19±6.41) compared to smokers (28.36±5.87) which was different at the 15% significance level.

MFV results were used to determine the patient's neurovascular response. For each of the three cycles, average MFV values ​​were calculated for the last 10 seconds with eyes closed and the first 40 seconds with eyes open. The neurovascular response was defined as the ratio of the difference between these two values ​​and the basal value and was expressed in percentages.

**Figure 5:**
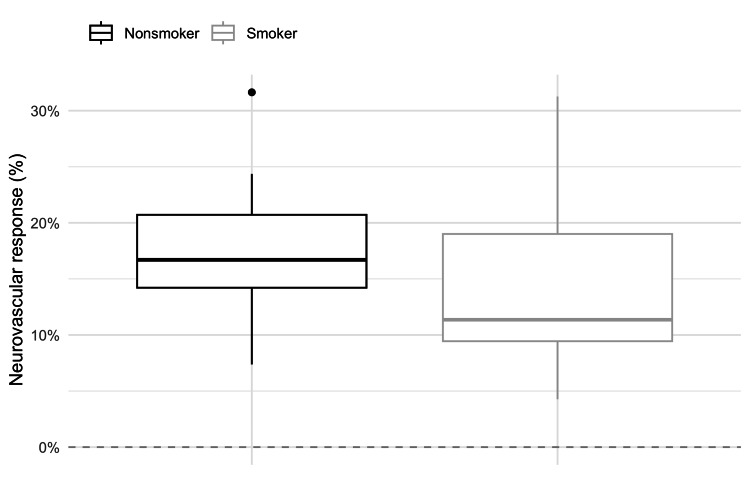
Total neurovascular response in nonsmokers and smokers

The total neurovascular response was calculated as the average value between the neurovascular response on the left and right sides. One-tailed t-test showed that nonsmokers had a more significant increase in MFV and neurovascular response than smokers (17.33%±0.06% versus 14.27%±0.08%, respectively). The observed differences were statistically significant at the 10% significance level, so we can conclude that nonsmokers have a better neurovascular response than smokers (Figure 5).

In all patients, the left and right side PI were measured in the last 10 seconds of the rest phase for all three cycles. The indices were averaged per cycle bilaterally and totally. One-tailed t-test showed that nonsmokers had lower levels of PI than smokers. These observed differences were statistically significant at a less than 10% significance level (Table 1).

**Table 1:** The result of the t-test for examining the differences in the pulsatility index in nonsmokers and smokers

Pulsatility index	M±SD	t	p
		-1.5	0.07
Nonsmokers	0.93±0.14		
Smokers	1.01±0.19		

Influence of smoking intensity on hemodynamic parameters

**Figure 6:**
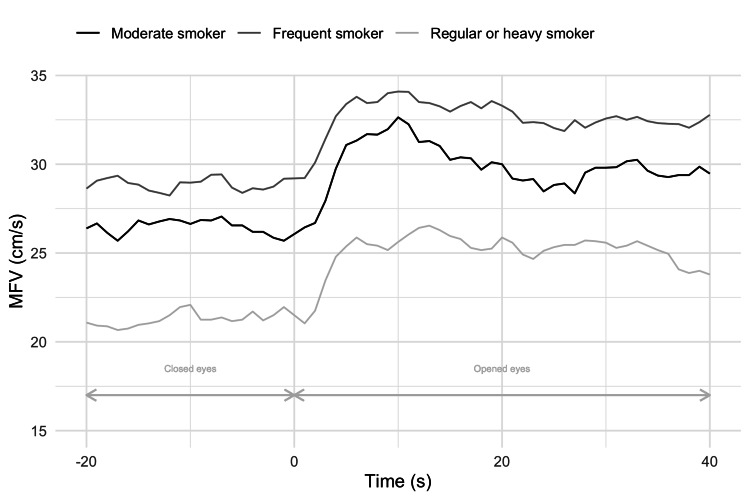
MFV values over time concerning smoking intensity MFV: mean blood flow velocity

MFVs were meticulously analyzed in relation to smoking intensity. To test the differences in MFV levels in smokers with regard to smoking intensity, we combined the last two groups, regular smokers and heavy smokers, into a new group: regular or heavy smokers. This was done with careful consideration, as there was only one patient in the heavy smoker group. MFV values ​​over time, with regard to smoking intensity, are shown in Figure 6.

Differences in MFV levels concerning smoking intensity were tested by ANOVA (Table 2). 

**Table 2:** Differences in MFV levels in smokers concerning smoking intensity (ANOVA) MFV: mean blood flow velocity; ANOVA: analysis of variance

MFV (cm/s)	M±SD	F	p
		3.00	0.08
Moderate smoker	28.75±20.79		
Frequent smoker	31.41±21.41		
Regular or heavy smoker	23.82±18.41		

The differences observed in MFV levels were statistically significant at the 10% significance level. Further post hoc analysis examined the differences between the groups (Table 3).

**Table 3:** Post hoc analysis of differences in MFV levels in smokers according to smoking MFV: mean blood flow velocity; *: one-tailed t-test

Group 1	Group 2	t*	p
Moderate smoker	Frequent smoker	-0.99	0.83
Moderate smoker	Regular or heavy smoker	1.63	0.07
Frequent smoker	Regular or heavy smoker	2.55	0.02

It can be concluded that the differences observed between frequent smokers and regular or heavy smokers were statistically significant at the significance level of 5%. Thus, regular or heavy smokers had lower MFV values ​​compared to frequent smokers. The same is true for moderate smokers versus regular or heavy smokers. Moderate smokers had higher MFV values ​​compared to regular or heavy smokers. However, the differences observed in that case were statistically significant at the 10% significance level. The differences between moderate and frequent smokers were not statistically significant.

ANOVA of the neurovascular response in relation to the intensity of smoking revealed no statistically significant differences between groups (p=0.41).

The relationship between the PI and the intensity of smoking was also investigated. ANOVA was used to test differences in smokers. There were no statistically significant differences in PI regarding the intensity of smoking (p=0.63).

## Discussion

Smoking is a leading primary and secondary risk factor for cardiovascular diseases that can be prevented. Our research was conducted on a population of healthy smokers without other known cardiovascular risk factors.

In the three-month prospective study, we enrolled 34 patients, 15 nonsmokers and 19 smokers. The average age of the subjects was 35±2 years. In general, the investigated and control groups were balanced except in sex where significantly more men were found in the group of smokers. This is also known in research on the prevalence of smoking [10].

Our research showed that in a sample of healthy subjects, total MFVs in smokers were statistically significantly lower (28.36±5.87 cm/s) than nonsmokers (30±6.41 cm/s). Earlier studies on CBF changes in smokers mainly used small samples in the 1980s, using different technologies [11,12]. Measurement of parameters after acute inhalation of smoking products has shown an increase in blood flow velocities in several studies [13,14]. Some studies have shown that CBF is not impaired in chronic smokers [15]. Still, in contrast, several studies have shown that regional CBF can be significantly reduced in chronic smokers, which is reversible several years after cessation of smoking [16,17]. How smoking alters CBF still needs to be fully understood. The relationship between chronic smoking and CBF is thought to arise from multiple interconnected pathways that may be impaired by oxidative stress, resulting in impaired endothelial function, nitric oxide (NO)-mediated vasodilation, hypocapnia, and vascular structural changes and damage [18].

Cortical activity, metabolism, and blood flow in the brain are closely interdependent. The visual stimulus promotes an increase in neuronal activity and metabolism and leads to a consequent increase in blood flow, which we call the neurovascular response.

In most studies on healthy subjects, there was an increase in MFV in PCA in the stimulation phase, depending on the complexity of the stimulus. Stimulation with a constant checkerboard led to a relative rise in MFV by 12.5%, and stimulation with color video film at 20-second intervals led to an increase of MFV by 43.4±14.9% [19,20].

We observed an increase in MFVs by 14.27±0.08% in smokers, compared to nonsmokers, who achieved a 17.33±0.06% rise. The visual stimulus was a static, neutral newspaper text on the screen. In a study by Olah et al., a similar visual paradigm was used to stimulate the visual cortex in 16 smokers and 16 nonsmokers. The maximum percentage of change in blood flow velocities after visual stimulation in smokers was 19±4% and in nonsmokers 30±3% [21]. These higher values ​​compared to ours can be explained by the analysis of maximum systolic blood flow velocities. In both of these studies, the analysis was performed after a few hours of abstinence, which indicated that smoking even then inhibits the visually evoked neurovascular response in otherwise healthy, younger smokers. Some studies went even further and showed that blood flow velocities and neurovascular response were significantly lower in smokers several months after quitting, which would indicate structural changes in blood vessels [22].

In our research, the PI was analyzed as a measure of microcirculation state. The PI in the last 10 seconds of the eyes-closed phase was statistically higher in smokers (1.01±0.19) compared to nonsmokers (0.93±0.14). A similar increase in PI and resistance index (RI) in smokers was found in other studies on chronic smokers [23].

None of these studies on smokers addressed the influence of smoking intensity on the measured parameters. The pattern between smoking intensity and hemodynamic parameters was observed but reached statistical significance in the smoking intensity and blood flow velocity relation. We observed that higher smoking intensity decreases blood flow velocities in smokers. These findings give us the rationale for further research on this topic on a more significant sample of smokers.

One of the possible limitations of this research is the non-duplex character of the test used, the reliability of which depends on the knowledge and experience of the examiner. Given that the research was conducted in an outpatient clinic, there was no possibility of checking certain blood parameters such as hemoglobin and carbon dioxide (CO_2_) concentration, which could possibly affect the measurement results. Another limitation of this study is the very small sample size of 34 subjects, thereby significantly reducing the statistical power of this study. That makes it difficult to generalize the results to a broader population. Consequently, it would be interesting to extend the research to multicentric higher-sample study on this topic in the future.

## Conclusions

Our research showed impaired hemodynamic parameters in young, otherwise healthy smokers that could indicate that chronic smoking deteriorates the vasodilatory mechanism in the cerebral vessels. Research in this area is of paramount importance because it enables clinicians to identify smokers particularly prone to vascular brain injury in various pathological conditions. Therefore, they should be subjected to tailored cessation programs and radical lifestyle changes.
